# Protective Effects of Perceived Control on Prescription Drug Misuse 10-years Later in the MIDUS Study

**DOI:** 10.1093/geroni/igab046.3124

**Published:** 2021-12-17

**Authors:** Sara Miller, David Almeida

**Affiliations:** Pennsylvania State University, University Park, Pennsylvania, United States

## Abstract

The current study examined if control beliefs predict prescription drug misuse (PDM) 10-years later and how problem drinking status moderates this relationship. PDM refers to taking medications without a prescription or in a manner not intended by the prescriber. Older adults are especially vulnerable to PDM due to drug sensitivity, comorbid health conditions, and high rates of polypharmacy. Participants were adults (n=2162, 56% female, mean age=54, range=30-84) who completed Waves 2 and 3 of the Midlife Development in the United States (MIDUS) study. At Wave 2, participants reported on two subscales of perceived control (personal mastery and constraints) and past 12-month problem drinking behaviors. At Wave 3, participants reported past 12-month PDM of five substances (painkillers, sedatives, stimulants, tranquilizers, and depression medications). Results indicated that at Wave 3, 10% of the sample reported misusing at least one prescription drug in the past year. Logistic Regression analysis revealed that problem drinking was associated with higher odds of PDM (p<0.001), and perceived control was associated with lower odds of PDM (p<0.05) after controlling for previous PDM and sociodemographic, health behavior, and health status covariates. However, there was an interaction effect such that perceived control was not protective for those individuals who engaged in problem drinking at Wave 2 (p<0.05). Future analyses will explore the meaning of this interaction. Identifying psychosocial protective factors, such as perceived control, predicting PDM will be critical for designing interventions that prevent the adverse consequences of PDM among this population.

